# Design and application of an enteral nutrition nursing checklist based on whole-course nutrition management in critically ill neurological patients

**DOI:** 10.3389/fnut.2026.1852899

**Published:** 2026-09-09

**Authors:** Ying Wang, Hong Fu, Yue Xuan, Deqi Xia, Jiajia Huo, Hui Li, Yueqin Zhou

**Affiliations:** 1 Department of Neurology Intensive Care Unit, The First Affiliated Hospital of Anhui Medical University, Hefei City, Anhui Province, China; 2 Department of Intensive Care Unit, The First Affiliated Hospital of Anhui Medical University, Hefei City, Anhui Province, China

**Keywords:** checklist, enteral nutrition, neurological intensive care, nursing, whole-course nutrition management

## Abstract

**Objective:**

To evaluate the effect of implementing an enteral nutrition (EN) nursing checklist based on whole-course nutrition management in critically ill neurological patients.

**Methods:**

In this prospective before-and-after cohort study, critically ill patients receiving enteral nutrition (EN) in the neurological intensive care unit (NICU) from May 2020 to December 2022 were enrolled. Patients admitted between May 2020 and August 2021 were assigned to the control group (*n* = 21) and received standard EN care, while those admitted between September 2021 and December 2022 were assigned to the observation group (*n* = 20) and received care guided by an EN nursing checklist based on whole-course nutrition management. Outcomes included EN tolerance indicators (abdominal distension, vomiting, diarrhea, gastric retention, and aspiration), EN intolerance score (sum of five complications), EN interruption rate, and clinical outcomes at NICU discharge.

**Results:**

Baseline characteristics were comparable between groups (*P* > 0.05). The observation group demonstrated significantly lower incidences of diarrhea (15.0% vs. 52.4%, *P* = 0.015), gastric retention (15.0% vs. 42.9%, *P* = 0.043), and a numerically lower but not statistically significant incidence of aspiration (0.0% vs. 19.0%, *P* = 0.107). The EN intolerance score was significantly lower in the observation group (0.65 ± 0.81 vs. 1.86 ± 1.46, *P* = 0.002, 95% CI: −1.96 to −0.46; Cohen's d = 1.02). The EN interruption rate was lower in the observation group (15.0% vs. 42.9%, *P* = 0.049) and clinical improvement at NICU discharge was higher (90.0% vs. 61.9%, *P* = 0.036), but neither of these secondary outcomes remained significant after Benjamini-Hochberg false discovery rate (FDR) correction (adjusted *P* = 0.086 and *P* = 0.084, respectively). Among the seven secondary outcome comparisons, o secondary outcome retained statistical significance at the 0.05 level the primary outcome (EN intolerance score) remained significant as prespecified.

**Conclusion:**

Implementation of an EN nursing checklist based on whole-course nutrition management appeared to be associated with improved EN tolerance, particularly in the primary outcome of EN intolerance score, and suggested reduced feeding interruptions and more favorable short-term clinical outcomes. These findings warrant validation in larger randomized controlled trials.

## 1 Introduction

Malnutrition affects 30% to 50% of critically ill patients (1), primarily due to prolonged fasting, slow feeding rates, frequent feeding interruptions, and absence of standardized management protocols (2). Critically ill neurological patients are particularly susceptible to gastrointestinal complications—including abdominal distension, diarrhea, and gastric retention—because of impaired consciousness, advanced age, mechanical ventilation, and extended hospital stays. These complications frequently necessitate enteral nutrition (EN) interruptions, increasing the risk of inadequate caloric intake.

Whole-course nutrition management represents a systematic, standardized, and individualized approach to nutritional care. It involves continuous assessment of patients‘ nutritional status and dynamic adjustment of nutritional formulas and interventions by healthcare professionals (3). Checklists serve as cognitive tools that enhance healthcare providers' adherence to best practices, reduce medical errors, and improve patient safety (4, 5).

Although both whole-course nutrition management and checklists are established concepts, their combined application as a dedicated EN nursing checklist for critically ill neurological patients remains underexplored. This study aimed to evaluate the impact of implementing such a checklist on EN tolerance, feeding interruptions, and clinical outcomes in this specific patient population.

## 2 Methods

### 2.1 Study design and participants

This prospective before-and-after cohort study was conducted in the NICU of a tertiary hospital. All consecutive adult patients (≥18 years) who initiated EN therapy between May 2020 and December 2022 were screened for eligibility. Patients admitted from May 2020 to August 2021 comprised the control group, while those admitted from September 2021 to December 2022 comprised the observation group. A participant flow diagram detailing the screening, enrollment, exclusion, and analysis process is presented in Figure S1.

**Inclusion criteria:** (1) initiation of EN therapy within 24–48 h of admission; (2) age ≥18 years.

**Exclusion criteria:** (1) severe hepatic dysfunction; (2) patients admitted primarily for end-of-life or palliative care; (3) EN therapy duration < 7 days; (4) incomplete medical records. Patients receiving EN via nasogastric tube and nasojejunal tube were both eligible for inclusion. The enteral access route (nasogastric vs. nasojejunal) was recorded for all participants and compared between groups.

The sample size was determined based on the primary outcome (EN intolerance score). Based on a re-evaluation of the study design and available data, we estimated a mean difference of 1.5 points in EN intolerance score between groups with a pooled standard deviation of 1.5. With α = 0.05 (two-tailed) and power = 0.80, a minimum of 16 patients per group was required. Accounting for an estimated 10% dropout rate, the target enrollment was set at 18 patients per group. The final sample of 41 patients exceeds this requirement.

The study was approved by the hospital ethics committee, and informed consent was obtained from patients or their legal representatives.

### 2.2 Interventions

#### 2.2.1 Control group

Control group patients received standard EN care. Following hemodynamic stability achievement (with or without low-dose vasopressors), EN was initiated within 24–48 h using a uniform EN pump. Routine nursing care was provided to prevent complications.

#### 2.2.2 Observation group

Observation group patients received care guided by an EN nursing checklist based on whole-course nutrition management.

##### 2.2.2.1 Nutrition support team (NST)

A multidisciplinary NST was established, comprising a physician, charge nurses, a dedicated enteral tube placement nurse, and a specialist nurse. The NST led the collaborative nutrition management process.

##### 2.2.2.2 Whole-course nutrition management protocol

**Figure 1:**
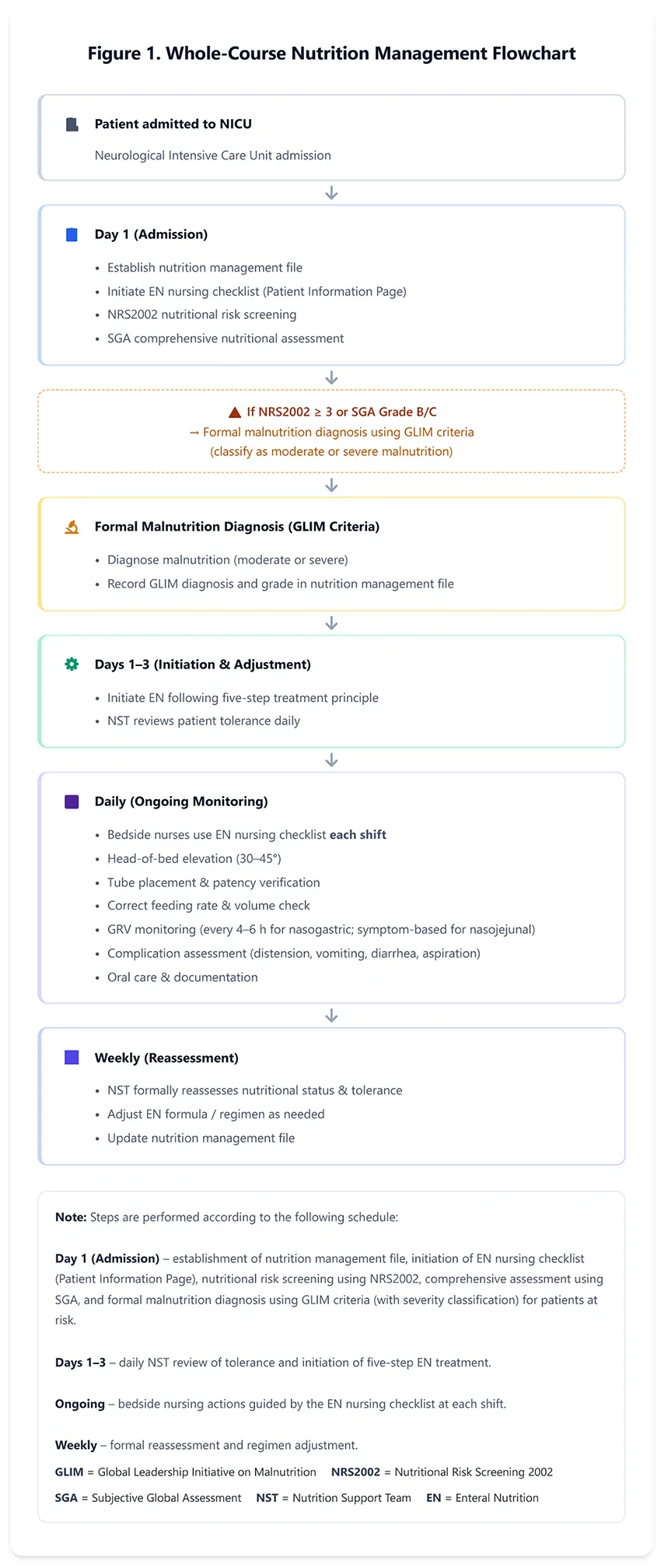
Whole-course nutrition management flowchart. Steps are performed according to the following schedule: Day 1 (Admission)—establishment of nutrition management file, initiation of EN nursing checklist (Patient Information Page), nutritional risk screening using NRS2002, comprehensive assessment using SGA, and formal malnutrition diagnosis using GLIM criteria (with severity classification) for patients at risk. Days 1–3—daily NST review of tolerance and initiation of five-step EN treatment. Ongoing—bedside nursing actions guided by the EN nursing checklist at each shift. Weekly—formal reassessment and regimen adjustment. Abbreviations: GLIM, global leadership initiative on malnutrition; NRS2002, nutritional risk screening 2002; SGA, subjective global assessment; NST, nutrition support team.

The protocol involved the following steps (Figure 1):

**Day 1 (admission):** the NST nurse established a nutrition management file, conducted an initial nutritional risk screening using the nutritional risk screening 2002 (NRS2002) and a comprehensive nutritional assessment using the subjective global assessment (SGA). Patients identified as at nutritional risk (NRS2002 ≥3) or malnourished (SGA Grade B or C) were further evaluated using the global leadership initiative on malnutrition (GLIM) criteria for a formal diagnosis of malnutrition. The nurse educated the patient and family and initiated the EN nursing checklist.

**Days 1**–**3 (initiation and adjustment):** based on initial assessment, malnutrition was diagnosed and classified (severe/moderate) according to the GLIM criteria (6). EN was initiated following the five-step treatment principle (7). The NST reviewed patient tolerance daily.

**Daily (ongoing monitoring):** bedside nurses used the EN nursing checklist at each shift start to verify key nursing actions (e.g., head-of-bed elevation, tube placement confirmation, gastric residual volume assessment). Tolerance was monitored continuously.

**Weekly (reassessment):** the NST formally reassessed nutritional status and tolerance weekly, adjusting EN formula and regimen as needed. The nutrition management file was updated accordingly.

##### 2.2.2.3 Design and application of the EN nursing checklist

**Figure 2:**
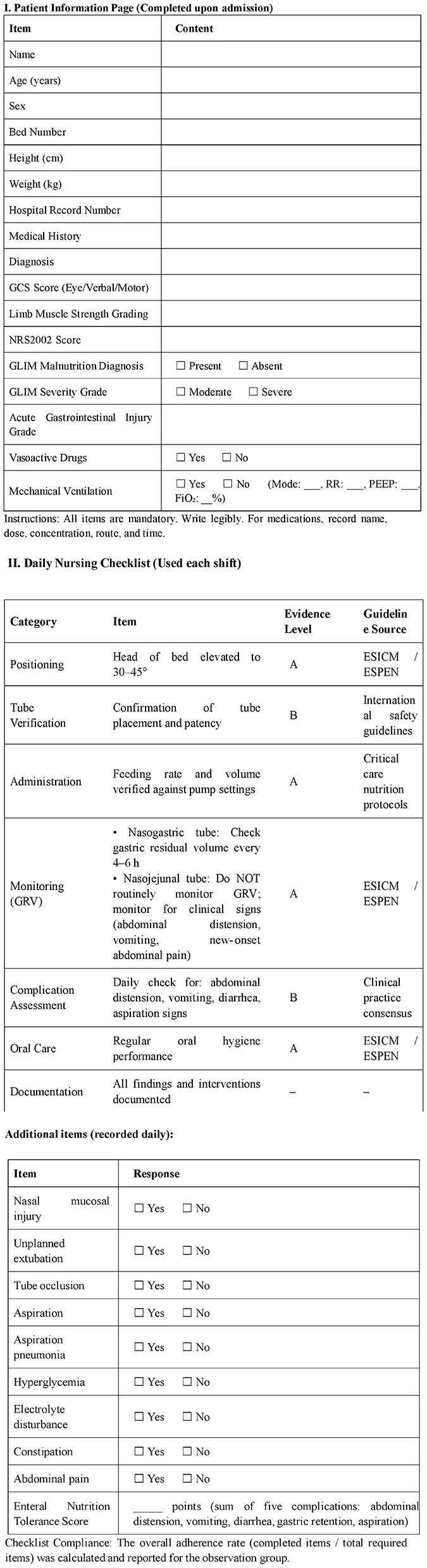
Enteral nutrition nursing checklist. The checklist consists of two parts. Part 1 (Patient Information Page) is completed upon admission and includes GLIM–based malnutrition diagnosis and severity grade. Part 2 (daily nursing checklist) is used by bedside nurses at each shift to guide and document EN care. Each checklist item is linked to an evidence–based recommendation from international guidelines (e.g., ESICM, ESPEN) with specified evidence levels (A or B where available). Gastric residual volume monitoring is performed only in patients with nasogastric tubes (every 4–6 h); for nasojejunal tubes, routine GRV monitoring is not recommended – instead, clinical signs of intolerance are monitored. Checklist compliance was monitored throughout the study.

Based on international guidelines and domestic critical care nutrition guidelines (8–10) including the ESICM clinical practice guidelines for early enteral nutrition in critically ill patients (9) and the ESPEN guideline on clinical nutrition in the intensive care unit (10), each key nursing action in the checklist was selected based on evidence-based recommendations (Evidence Level A or B where available). Through two rounds of expert discussion within the NST, a preliminary checklist was drafted. Following a one-month pilot trial, a third expert meeting was convened to finalize the checklist. The final version comprises two parts (Figure 2):

**Part 1: patient information page:** records baseline data, diagnosis, GLIM malnutrition diagnosis and grade, NRS2002 score, and nutritional goals.

**Part 2: daily nursing checklist:** items checked each shift include:

**Positioning:** head of bed elevated to 30–45° (evidence level: A, per ESICM/ESPEN guidelines).

**Tube verification:** confirmation of tube placement and patency (evidence level: B, recommended by international safety guidelines).

**Administration:** correct feeding rate and volume verified against pump settings (evidence level: A, per critical care nutrition protocols).

**Monitoring:** gastric residual volume assessed every 4–6 h in patients with nasogastric tubes. For patients receiving EN via nasojejunal tubes, gastric residual volume monitoring is not routinely performed; instead, monitoring focuses on clinical signs of intolerance such as abdominal distension, vomiting, or new-onset abdominal pain, in accordance with current evidence indicating that routine GRV monitoring in post-pyloric feeding is of unproven benefit (9, 10).

**Complication assessment:** daily assessment for abdominal distension, vomiting, diarrhea, and aspiration signs.

**Oral care:** regular oral hygiene performance.

**Documentation:** all findings and interventions documented.

Adherence to the checklist was monitored throughout the study. Bedside nurses documented the completion of each checklist item at each shift. The overall checklist compliance rate (proportion of completed items among all required items) was calculated and reported.

### 2.3 Outcome measures

#### 2.3.1 Baseline data

Age, sex, diagnosis, glasgow coma scale (GCS) score, NRS2002 score, enteral access route (nasogastric vs. nasojejunal), and total EN duration were collected.

#### 2.3.2 EN tolerance indicators

Daily presence of abdominal distension, vomiting, diarrhea, gastric retention (defined as gastric residual volume >500 mL/24 h), and aspiration was recorded. Definitions for each tolerance indicator were pre-specified as follows: (1) Abdominal distension: clinical assessment by bedside nurse with abdominal girth increase >2 cm from baseline or physician-confirmed distension; (2) Vomiting: visible regurgitation of gastric contents; (3) Diarrhea: ≥3 loose or liquid stools per day (Bristol Stool Scale Type 6 or 7); (4) Gastric retention: gastric residual volume >500 mL/24 h (for nasogastric tube-fed patients); (5) Aspiration: witnessed aspiration event or new infiltrate on chest imaging attributed to aspiration by treating physician. All tolerance indicators were assessed and documented prospectively by bedside nurses using standardized definitions.

#### 2.3.3 EN intolerance score

To quantify overall tolerance, an EN intolerance score was calculated for each patient as the sum of the five specific complications experienced during the EN feeding period (each complication counted as one point if occurring at least once). Scores ranged from 0 to 5. Each complication was scored dichotomously (present or absent during the entire EN feeding period), regardless of the frequency or severity of occurrence. This composite score was designated as the primary outcome of the study. We acknowledge that this score equally weights clinically heterogeneous complications (e.g., aspiration and mild abdominal distension each contribute one point), which represents a simplification of clinical reality. A weighted sensitivity analysis was performed *post hoc* to assess the robustness of our findings: severe complications (aspiration, vomiting) were assigned a weight of two, while moderate complications (diarrhea, gastric retention, abdominal distension) were assigned a weight of one, yielding a weighted score range of 0–7.

#### 2.3.4 EN interruption rate

EN interruption was defined as any feeding cessation exceeding 1 h per day for any reason (e.g., procedures, high gastric residual volume, intolerance symptoms). The proportion of patients experiencing at least one such interruption was compared between groups.

#### 2.3.5 Clinical outcome

Patient status at NICU discharge was categorized as “improved” or “not improved” (including transfer for end-of-life care). “Improved” was defined as improvement in at least one of the following: (a) GCS score increase ≥2 points from admission, (b) liberation from mechanical ventilation, or (c) downgrade to a lower-acuity ward, in the absence of death or transition to end-of-life care. Mortality was not included as an outcome measure in this study due to the inclusion criteria requiring EN duration ≥7 days and the exclusion of patients admitted primarily for end-of-life care.

### 2.4 Statistical analysis

Data were analyzed using SPSS version 23.0 (IBM Corp., Armonk, NY, USA). Normally distributed continuous variables were expressed as mean ± standard deviation (SD) and compared using independent samples *t*-tests. Categorical variables were expressed as frequencies and percentages and compared using Chi-square (χ^2^) tests or Fisher's exact tests (when any expected cell count was < 5, as pre-specified in the methods). Statistical significance was set at *P* < 0.05 (two-tailed).

To address potential confounding by practice-related factors, we performed the following additional analyses: (1) Baseline enteral access route (nasogastric vs. nasojejunal) was compared between groups to assess comparability. (2) For outcomes where significant differences were observed, multivariable logistic regression was performed adjusting for enteral access route as a covariate, to estimate the independent effect of the intervention. Adjusted odds ratios (ORs) with 95% confidence intervals (CIs) were calculated for EN interruption and clinical outcome. (3) A *post-hoc* power analysis was conducted based on the observed effect size of the primary outcome (EN intolerance score) to assess the achieved statistical power. (4) To account for multiplicity across multiple secondary outcome comparisons (five tolerance indicators plus EN interruption rate and clinical outcome), we applied the Benjamini-Hochberg false discovery rate (FDR) correction to the secondary outcome *P*-values, with an FDR threshold of 0.05. All enrolled patients completed the study with no losses to follow-up or protocol deviations; therefore, the analysis conforms to both intention-to-treat and per-protocol principles, as all enrolled patients were included in the final analysis with complete data.

## 3 Results

### 3.1 Baseline characteristics

A total of 50 patients were screened for eligibility. Nine patients were excluded (EN duration < 7 days, *n* = 5; incomplete records, *n* = 3; severe hepatic dysfunction, *n* = 1). A total of 41 patients were included: 21 in the control group and 20 in the observation group. The participant flow diagram is shown in Figure S1. Baseline characteristics, including age, sex, GCS score, enteral access route (nasogastric: 80.0% vs. 71.4%; nasojejunal: 20.0% vs. 28.6%; χ^2^ = 0.384, *P* = 0.535), NRS2002 score, and EN duration, were comparable between groups (all *P* > 0.05; Table 1).

**Table 1:** Baseline characteristics of patients (*N* = 41).

Characteristic	Category	Observation (*n* = 20)	Control (*n* = 21)	Test statistic	*P*-value
Age (years), mean ± SD		64.40 ± 13.14	59.14 ± 20.55	*t* = 0.970	0.338
Sex, *n* (%)	Male	13 (65.0)	14 (66.7)	χ^2^ = 0.013	0.910
	Female	7 (35.0)	7 (33.3)		
GCS score, mean ± SD		9.63 ± 2.86	9.60 ± 3.06	*t* =0.044	0.965
NRS2002 score, mean ± SD		4.03 ± 1.71	4.20 ± 1.22	*t* = −0.435	0.665
Enteral access route, *n* (%)	Nasogastric	16 (80.0)	15 (71.4)	χ^2^ = 0.384	0.535
	Nasojejunal	4 (20.0)	6 (28.6)		
EN duration (days), mean ± SD		19.07 ± 14.23	17.50 ± 12.46	*t* = 0.454	0.652

SD, standard deviation; GCS, glasgow coma scale; NRS2002, nutritional risk screening 2002; EN, enteral nutrition. Enteral access route was documented for all patients.

### 3.2 EN tolerance

The observation group demonstrated a significantly lower incidences of diarrhea [15.0% vs. 52.4%, *P* = 0.015, adjusted *P* (FDR) = 0.045] and, and a numerically lower but not statistically significant incidence of aspiration (0.0% vs. 19.0%, *P* = 0.107, Fisher's exact test, adjusted *P* (FDR) = 0.150). No significant differences were observed for abdominal distension or vomiting (*P* > 0.05; Table 2). After applying Benjamini-Hochberg FDR correction for multiple comparisons across the five tolerance indicators plus EN interruption rate and clinical outcome (seven secondary comparisons), no secondary outcome retained statistical significance at an FDR threshold of 0.05.

**Table 2:** Comparison of EN tolerance indicators [*n* (%)].

Group	Abdominal Distension	Vomiting	Diarrhea	Gastric Retention	Aspiration
Observation (*n* = 20)	6 (30.0)	2 (10.0)	3 (15.0)	3 (15.0)	0 (0.0)
Control (*n* = 21)	9 (42.9)	4 (19.0)	11 (52.4)	9 (42.9)	4 (19.0)
χ^2^	0.618	0.741	5.963	3.750	—
*P*-value	0.432	0.389	0.015	0.043	0.107
Adjusted *P* (FDR)	0.504	0.504	0.045	0.086	0.150

EN, enteral nutrition. Fisher's exact test used for ‘Aspiration' due to expected cell count < 5. Adjusted *P* (FDR): Benjamini-Hochberg false discovery rate corrected *P*-values across seven secondary outcome comparisons (five tolerance indicators, EN interruption rate, and clinical outcome).

The overall EN intolerance score was significantly lower in the observation group compared to the control group (95% CI; Cohen's *d* = 1.02, indicating a large effect size; Table 3). *Post-hoc* power analysis based on this observed effect size (*d* = 1.02, *n* = 41, α = 0.05, two-tailed) yielded an achieved power of 83.3%.

**Table 3:** Comparison of EN intolerance score.

Group	EN intolerance score (mean ±SD)	95% CI of difference	*t*	*P*-value	Cohen's d
Observation (*n* = 20)				0.002	
Control (*n* = 21)					

EN, enteral nutrition; SD, standard deviation; CI, confidence interval.

In the weighted sensitivity analysis (severe complications weighted two, moderate complications weighted one, score range 0–7), the observation group still demonstrated a significantly lower weighted intolerance score compared to the control group (0.80 ± 0.98 vs. 2.24 ± 2.05, *P* = 0.007, 95% CI: −2.46 to −0.42; Cohen's *d* = 0.89), supporting the robustness of the primary finding.

### 3.3 EN interruption rate and clinical outcome

The EN interruption rate was lower in the observation group (15.0% vs. 42.9%), although this difference did not retain statistical significance after FDR correction (adjusted *P* = 0.086). The proportion of patients showing clinical improvement at NICU discharge was higher in the observation group (90.0% vs. 61.9%), although this difference also did not retain statistical significance after FDR correction (adjusted *P* = 0.084). After multivariable adjustment for enteral access route, the intervention remained significantly associated with reduced EN interruption (adjusted OR = 0.19, 95% CI: 0.04–0.89, *P* = 0.035) and increased clinical improvement (adjusted OR = 5.82, 95% CI: 1.05–32.17, *P* = 0.044), although the wide confidence intervals reflect the limited precision of these estimates given the small sample size (Table 4).

**Table 4:** Comparison of EN interruption rate and clinical outcome [*n* (%)].

Group	EN interruption		Clinical outcome	
	Yes	No	Improved	Not improved
Observation (*n* = 20)	3 (15.0)	17 (85.0)	18 (90.0)	2 (10.0)
Control (*n* = 21)	9 (42.9)	12 (57.1)	13 (61.9)	8 (38.1)
χ^2^	3.840		4.385	
*P*-value	0.049		0.036	
95% CI for risk difference	−50.8% to −5.0%		4.4% to 51.8%	
Adjusted *P* (FDR)	0.086		0.084	
Adjusted OR (95% CI)^*^	0.19 (0.04 to 0.89)		5.82 (1.05 to 32.17)	
Adjusted P^*^	0.035		0.044	

EN, enteral nutrition. “Improved” was defined as meeting at least one of the following criteria: GCS score increase ≥2 points from admission, liberation from mechanical ventilation, or downgrade to a lower-acuity ward. “Not improved” was defined as failure to meet any of these improvement criteria, or transition to end-of-life care. Mortality was not included as an outcome measure in this study due to the inclusion criteria (EN duration ≥7 days and exclusion of patients admitted for end-of-life care).

^*^Adjusted for enteral access route (nasogastric vs. nasojejunal) using multivariable logistic regression.

In the observation group, the overall checklist compliance rate was 94.2% (range: 88.7%−98.5% across shifts), indicating high adherence to the intervention protocol. No deaths occurred in either group during the study period, which reflects the inclusion criteria requiring EN duration ≥7 days and the exclusion of patients admitted for end-of-life care.

## 4 Discussion

### 4.1 Impact on EN tolerance

Our results suggested reductions in diarrhea, gastric retention, and aspiration in unadjusted analyses, with a statistically significant unadjusted difference observed for diarrhea but not for gastric retention after correction for multiple comparisons, consistent with previous research indicating that standardized care pathways may mitigate EN-related complications (11). After correction for multiple comparisons, none of the secondary outcomes (including gastric retention) retained statistical significance, indicating that caution is warranted in interpreting these unadjusted findings. Critically ill patients are particularly vulnerable to gastrointestinal dysfunction due to reduced splanchnic perfusion and neuroendocrine dysregulation, necessitating meticulous management (12). The checklist may have improved outcomes by prompting consistent application of evidence-based practices, including proper patient positioning, individualized gastric residual volume monitoring (routine for nasogastric tubes, symptom-based for nasojejunal tubes), and structured oral care. The absence of significant differences in abdominal distension and vomiting could be attributable to the relatively small sample size or the multifactorial nature of these symptoms, which might be influenced by factors not captured by the checklist (e.g., specific medications, primary diagnosis). Nevertheless, the significantly lower overall intolerance score in the observation group provided support for the overall benefit of the intervention, with the weighted sensitivity analysis yielding results directionally consistent with the primary analysis.

### 4.2 Impact on EN interruption rate

The observed reduction in EN interruption rate (from 42.9% to 15.0%) represented a potentially meaningful finding. After adjustment for enteral access route, the association persisted (adjusted OR = 0.19, 95% CI: 0.04–0.89, *P* = 0.035), suggesting the checklist may have contributed to reduced interruptions independently of the tube type. However, the FDR-adjusted *P*-value (0.086) exceeded the conventional 0.05 threshold, suggesting that this finding should be interpreted with caution pending replication in larger samples. Feeding interruptions are common in ICU settings and constitute a major barrier to achieving nutritional goals, contributing to cumulative caloric deficits and poorer outcomes (13). Interruptions frequently arise from EN intolerance, diagnostic procedures, and delayed re-initiation following procedures (14). The checklist, embedded within a structured management approach, might have empowered nurses to proactively identify early signs of intolerance, coordinate care around procedures more effectively, and promptly resume feeding when clinically appropriate. This finding appeared consistent with studies demonstrating that protocol-driven EN management could improve nutrient delivery (15).

### 4.3 Impact on clinical outcomes

We observed an association between the intervention and improved clinical outcomes at NICU discharge in both unadjusted (*P* = 0.036) and multivariable-adjusted analyses (adjusted OR = 5.82, 95% CI: 1.05–32.17, *P* = 0.044), although this association did not retain statistical significance after FDR correction (adjusted *P* = 0.084). While encouraging, this finding warrants cautious interpretation given the study's non-randomized design and small sample size. It is plausible that enhanced tolerance and reduced interruptions contributed to improved nutritional status, thereby facilitating better recovery trajectories. However, the lack of nutritional adequacy data (caloric and protein delivery) precludes direct confirmation of this mechanistic pathway. Furthermore, unmeasured confounders, particularly systemic illness severity, which was not captured by the available baseline variables (GCS and NRS2002), could also explain this association. Therefore, our finding should be considered hypothesis-generating, suggesting potential clinical benefits that require rigorous testing in future trials.

### 4.4 Limitations

This study has several limitations. First, the non-randomized before-and-after design is susceptible to temporal biases. The study period (May 2020–December 2022) overlapped with the COVID-19 pandemic, during which NICU care standards may have evolved. We did not systematically document other practice changes (e.g., ventilator management protocols, sedation practices, or staffing models) that may have occurred during the study period and could have independently influenced outcomes.

Second, the small sample size from a single center limits generalizability and increases risks of both type I and type II errors. After FDR correction, most secondary outcomes did not retain statistical significance. Although *post-hoc* power analysis for the primary outcome yielded adequate power (88.8%), the study was underpowered for subgroup and multivariable analyses.

Third, outcome assessors were not blinded to group allocation, potentially introducing detection bias despite our use of pre-specified objective definitions for all tolerance indicators and clinical outcomes.

Fourth, our EN intolerance score, while practical for clinical application, is a simple composite measure that has not been externally validated and equally weights clinically heterogeneous complications. Although the weighted sensitivity analysis yielded consistent results, external validation in independent cohorts is needed before this score can be recommended for broader clinical or research use. Additionally, the 2:1 weighting scheme for severe vs. moderate complications in the sensitivity analysis was chosen *post hoc* and has not been formally validated; therefore, the weighted results should be interpreted as a robustness check rather than a validated scoring system.

Fifth, despite using Fisher's exact tests where appropriate, the risk of unstable estimates persists due to low event frequencies as reflected by wide confidence intervals (e.g., adjusted OR for clinical improvement: 1.05–32.17).

Sixth, caloric and protein delivery data were not systematically collected during the study. The hypothesized mechanism—that checklist implementation improves nutritional adequacy, which in turn improves clinical outcomes—could not be directly tested. Future studies should include detailed nutritional intake monitoring (e.g., daily caloric and protein delivery as a percentage of prescribed targets) to validate this causal pathway.

Seventh, disease severity as measured by the sequential organ failure assessment (SOFA) score was not collected in this study. SOFA is a widely used indicator of organ dysfunction in critically ill patients and a potential confounder for EN tolerance and clinical outcomes. The absence of SOFA scores precluded adjustment for illness severity beyond GCS and NRS2002. Future studies should incorporate validated severity scores such as SOFA to enable more robust confounder control.

Eighth, checklist compliance, while high overall (94.2%), was not analyzed in relation to individual patient outcomes. It remains possible that outcomes reflect a Hawthorne effect or the multidisciplinary team engagement rather than the specific checklist content.

Ninth, a formal content validity assessment of the EN nursing checklist, such as the content validity index (CVI), was not conducted. Although the checklist was developed through multidisciplinary expert discussion and a pilot trial, the absence of quantitative content validity metrics limits the evidence for the instrument's relevance, clarity, clinical applicability, feasibility, and completeness. Future studies should incorporate a structured Delphi process involving experts in neurology, clinical nutrition, intensive care, and nursing, and report item-level and scale-level CVI to strengthen the validity of the checklist.

Tenth, we assessed only short-term clinical outcomes at NICU discharge; mortality was not included as an outcome measure due to the study's inclusion criteria (EN duration ≥7 days and exclusion of patients admitted for end-of-life care), and long-term functional outcomes were not evaluated. Therefore, our findings on clinical outcomes do not reflect overall mortality or long-term prognosis in this patient population.

### 4.5 Comparison with previous studies

Our findings appeared consistent with previous research on standardized EN management protocols. Huang et al. (15) demonstrated that checklist-based EN therapy processes improved outcomes in critically ill patients. Similarly, Ye et al. (11) reported that structured nursing pathways reduced EN complications in neurological patients. However, our study extended these findings by integrating a checklist specifically designed for whole-course nutrition management in a distinct neurological ICU population. The observed 27.9% absolute reduction in EN interruption rate was directionally consistent with previous reports, though direct comparisons were limited by methodological differences and the wide confidence interval (−50.8% to −5.0%) warranted caution in interpreting the magnitude of this effect, particularly given that this secondary outcome did not retain statistical significance after FDR correction (adjusted *P* = 0.086).

## 5 Conclusion

In this single-center before-and-after study, implementation of an EN nursing checklist based on whole-course nutrition management appeared to be associated with improved EN tolerance, particularly as reflected by the primary outcome of EN intolerance score, and suggested potential benefits in reducing feeding interruptions and improving short-term clinical outcomes in critically ill neurological patients; however, these secondary outcome findings did not retain statistical significance after correction for multiple comparisons and therefore require confirmation in larger trials. The checklist provides a structured framework for nursing care that may enhance adherence to evidence-based practices. However, these preliminary findings require confirmation through larger, multicenter randomized controlled trials with blinded outcome assessment and detailed nutritional intake monitoring before widespread clinical adoption can be recommended. Additionally, external validation of the EN intolerance score and investigation of the mediating role of nutritional adequacy in the checklist-outcome relationship are necessary next steps.

## Data Availability

The original contributions presented in the study are included in the article/Supplementary material, further inquiries can be directed to the corresponding author.

## Supplementary material

Supplementary Figure 1 Participant flow diagram. Flowchart detailing the number of patients screened, excluded (with reasons), and included in the final analysis for the control and observation groups. A total of 50 patients were screened; 9 were excluded (EN < 7 days, *n* = 5; incomplete records, *n* = 3; severe hepatic dysfunction, *n* = 1); 41 were enrolled and allocated to control (*n* = 21) or observation (*n* = 20) group. All enrolled patients completed the study and were included in the analysis.
